# Four Letters with Replies

**Published:** 1877-10

**Authors:** 


### Our Correspondence.

KING'S FERRY, N. Y., July 6, 1877.

DR. UP DE GRAFF:

I am thirty-five years of age, and work at the milliner business. My eyes have been steadily failing me for about six years, growing more and more near-sighted, compelling me to procure stronger glasses every year. During the past month have used them more than usual, and I find my vision is becoming very dim. I am forced to stimulate my eyes with equal quantities of rose-water, brandy and tea, then wear a cloth over them nights wet with pretty strong salt water. I fear that they will suddenly fail me, or that I will become blind. Can you tell me what the trouble is? A. M. C.

You have what is known to oculists as *progressive myopia.* You should abandon work for a season, and be treated by some competent oculist, else will blindness ensue, sooner or later.

---

MIDWAY, ALA., Sept. 13, 1877.

DR. UP DE GRAFF:

I have a little patient—a girl, aged ten, who has commencing Pott's disease of the spine. It is with extreme difficulty that she can sit up, the pain in the back being so severe. I notice that a curvature is becoming apparent, and have recommended the parents to take her to your Institute for treatment. The distance is so great, however, that it seems quite an undertaking, and I have resolved to write to you first to ascertain whether she could be successfully treated at home by myself under your advice? Please advise me in the matter. J. D. S., M. D.

Apply a gypsum splint, which will give instant relief, and enable the child to run about. Have mailed you full directions for its application.

---

TYRONE, PA., Sept 5, 1877.

DR. UP DE GRAFF:

Your agent called last week and sold me a pair of spectacles, and said he would exchange them when he called again, if they did not suit. When will he be around again? I want to exchange them for a pair that I can see from. J. D.

Do not know *when* he will call again. When he *does* come, however, kick him and his miserable spectacles out doors and tell him that he is a swindler.

For the hundreth time, or thereabout, we state that we have no agents,—do not require any, neither do we deal in spectacles, nor encourage traveling quacks of *any* sort.

Take our advice, and never purchase anything from peddlers. You can always do better of regular dealers in your own city or village.

---

### MOUNTAIN AND CANON.

DENVER, COLORADO, September, 1877.

Cheyenne mountain is not among the great ones of the earth as regards hight. It is neither the first, second nor fourth in that list of honor; but, though standing beside Pike's Peak, which holds its head four thousand feet higher, it does not look less grand. Always over its "rock ribbed and thunder riven" form it draws the purple mist-garment with regal pride. Its canon, though seen after the surprise and wonder the first sight of such rocky defiles had been expended on other canons, is to me the finest I have seen.

The mountain's base is almost equally distant, five miles, from Colorado Springs and Manitou. At the latter place are all the springs that have given the former its celebrity. The soda spring is the finest I ever tasted, having a real soda water sparkle and flavor and a delicious coldness. The iron spring is certainly as good of its kind, but the soda spring is vitalized water, if such ever flowed from earth.

There are as good and expensive hotels here as one could ask for, and as splendid camping grounds as there could have been in the garden of Eden. The hotels are as full of the *elite* from all parts of this, and from many places in other countries, as are the surrounding camps of students and divines. Indeed, almost the only thing in the tone of western society which I have found to admire, is the general enthusiasm for out-door life, and the popularity of camping out for a month or more in summer. Ladies and all quite exult in roughing it. The unfailing glorious brightness of the sky, the absence of heavy dew, the cool mountain air, making camping a much less intricate matter than in the East. Water is the only distress cry here. The length of one's last ditch is the proudest matter of discussion

No one should come to Colorado without seeing the plains from the road that climbs Cheyenne mountains. The more noted wonders of the garden of the gods, Monument Park, Ute Pass, etc., have none of them just that kind of beauty which this mountain road leaves in perspective behind and in advance of the traveler. At every sharp angle of the road, which, for nearly nine miles, tacks up the mountain, a glowing, boundless glory of shining plains and sky are in the opposite direction—solemnity of piled mountain peaks with deep purple shades between. To the right, all sunshine; to the left, all shade; the one all peace and freedom, the other all forbiddingness of rocky steeps.

The most inspiring object in sight is a grand peak, surmounted by a castle-like rock, whose resemblance to the Fortress of St. Angelo is remarkable, and the day it led my wondering fancy toward it for hours, a little white cloud, floating up from its crowning turret, seemed the spirit of the great bronze angel of the old fortress on the Tiber.

In the return drive at evening the plains seem more majestic in expanse than ever. My driver, a lately arrived Englishman, remarked, looking eastward over them: "Ess, ye cood put the whole o' England doon there, wi'out settin't on a 'ouse."

There is something to me in the coming of night over these plains inexplicably solemn. Never any scene in nature, by night or day, not excepting twilight in the deep woods, the entrance into sun-forbidden-caves, or sullen sky over stormy water, has the solemnity of the coming of the dark over these land wastes.

The sky is always wonderfully bright, and the dark seems to breathe out of earth, or, like the garments of the genii of shade, to trail over it; and before one seems to stand the spirit of silence with a finger on her lip and another pointing to earth, in which are so many graves. * * * But under the throne of the mountain, Cheyenne keeps her own treasures, in a three mile canon leading to the seven falls.

A sufficiently reckless or sufficiently careful driver can make three-fourths of a mile, or a little more, of the way with a carriage. For the rest there is only a broken trail. I long to tell about the next two miles, but they are beyond words. Only two colors, red-brown and green,—a leaf and a stone multiplied. I could only repeat to myself, "What a piece of carving!" From the tops of the Gibralter-like walls of Pulpit Rock and the Dome of St. Peter's, which face each other in equal pride and dignity, every foot in their added thousands has its own hewn stone of druidic grandeur, down to the very leaf that holds a motionless shadow of its matchless shape upon some fallen boulder.

Studies for every chiseler, from the hewer of monumental shafts to the lace-like carver on an ivory fan, are in every foot of the way, being far over head or under the hand you lay on the rock at your side.

The mountain brook, which must be crossed more than a dozen times in making the ascent of the canon, has scarcely lost the coldness or crystal beauty of the snowflakes of which it is made. Each drop is the kiss of a sunbeam on a snowflake. Truly, I have seen Tiffany's counters under the gaslight, but never anything so bewitchingly, royally bright as these mountain brooks.

The seven falls, which should give their name to the canon, come leaping down a steep, winding stairway of rocks, the last of them only visible from below. A very rough climb over shaling rocks is necessary to gain the summit, from which they can be looked down upon, and from which they are rarely beautiful. This canon and Pike's Peak are rivals in my memory of Colorado glories.

On the following day I joined an excursion to the summit of Veta Pass, the highest point reached by railroad on the continent, crossing the Sangre de Christo range. The difficulties of engineering over such mountains have resulted in an average grade of 160 feet to the mile, and curves which quite outdo the Horse Shoe of the Pennsylvania Central, being in fact but thirty degrees frequently. I believe the gentleman was riding on the engine who said, "the track looked like a couple of dissipated angle worms out on a climb." The engineers of Clear Creek canon had to work harder for their success, perhaps, but the success is no more signal than this. As the ascent of Damp mountain is made by a road whose next curve we can see directly above us hundreds of feet, passengers get off the creeping train and climb over the mountain, and have time to gather flowers before the train reaches them at the next curve.

At Garland, the then terminus of the road, we found two hundred of the newest possible houses, where two weeks before there was not one. These towns follow the temporary railroad termini like a crowd of boys the organ-grinder's halts, and there the employes drop their money into the Mexican dance houses, drinking saloons and gambling houses. Only a Bret Harte could see any romance in these settlements. God pity the women, yes, the Eastern ladies, who have to live in them.

A. A. GLEASON, M. D.

